# Giant Anterior Neck Lipoma with Bleeding Pressure Ulcer in an Elderly Man: A Rare Entity

**Published:** 2017-09

**Authors:** Gaurav Jain, Ila Tyagi, Leela Pant, Namrata Nargotra

**Affiliations:** Department of Pathology, NDMC Medical College, Hindu Rao Hospital,Malkagunj, New Delhi, 110002, India

**Keywords:** Lipoma, Anterior neck, Giant, Ulcer, Large

## Abstract

Giant lipomas are benign soft tissue tumors found rarely in the neck and are still rarer in the anterior part of the neck. A 70-year-old male patient was presented with a huge swelling measuring 35 cm in maximum dimension, in the front of the neck, reaching up till the umbilicus. The swelling was painless, slow growing and acquired the huge size in approximately 20 years. Ultrasound and CT scan findings were suggestive of a soft tissue lesion. Fine needle aspiration cytology yielded mature adipose tissue fragments. A complete surgical removal of the mass was done which on gross examination, measured 32 cms in longest diameter and weighed 2500 grams. Diagnosis of giant anterior neck lipoma with pressure ulcer was confirmed on histopathology. We described a case of excessively large lipoma of anterior neck, which is the largest anterior neck lipoma with pressure ulcer reported till date.

## INTRODUCTION

Lipomas are common benign mesenchymal tumors usually found in the subcutaneous tissue but occurring infrequently in the head and neck with anterior neck being the rarest site. A lipoma is considered to be giant when it is greater than 10 cm in length (in any dimension) or weighs over 1000 grams. A large neck mass (>10 cm) with a rapid growth rate should raise concerns about a possible malignancy.^1^^-^^5^

## CASE REPORT

**Fig. 1:**
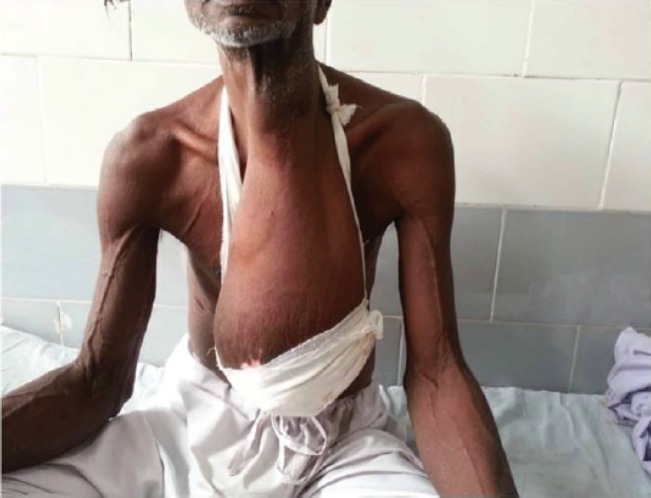
Clinical photograph of the patient showing huge swelling arising anteriorly from the neck more so on the left side and hanging up to the umbilicus

A 70-year-old male patient was presented to us with a huge swelling measuring 35 cms in greater dimension arising anteriorly from the neck more on the left side which was hanging up to the umbilicus (Figure 1). The swelling was painless, slow growing and had acquired the present size in approximately 20 years. There was no history of treatment in the past. Patient was completely asymptomatic, except for ulceration of the overlying skin. On palpation, the swelling was smooth, painless and non-tender. Skin over the swelling was stretched, shiny and showed ulceration over the lower most part. There was no lymphadenopathy or any other palpable mass. 

ENT examination of the patient was normal. Thyroid hormonal evaluation showed no abnormality. On ultrasound, the “mass” was iso-echoic with subcutaneous fat. It laid in front of the left lobe of the thyroid which was normal in size and echogenicity and moved freely with swallowing under the mass. The mass appeared to have an ill-defined capsule with several thin echogenic septa seen coursing through the substance of the mass. The ultrasound findings were suggestive of a soft tissue lesion. Computed tomography (CT) scan of the neck showed a hypodense mass in the left side of the neck with fat density, measuring 17×9 cm with multiple internal septations. 

It originated in the left anterior triangle of the neck and extended up to the upper chest cavity. The mass was seen to be causing mild pressure effects with displacement of left parapharyngeal and oropharyngeal spaces. The trachea and the major vessels were displaced to the right and both the lobes of the thyroid seemed normal. Findings were suggestive of a soft tissue lesion possibly lipoma. A fine needle aspiration was performed and samples were taken from multiple levels of the swelling for adequate and representative cytological material. 

**Fig. 2:**
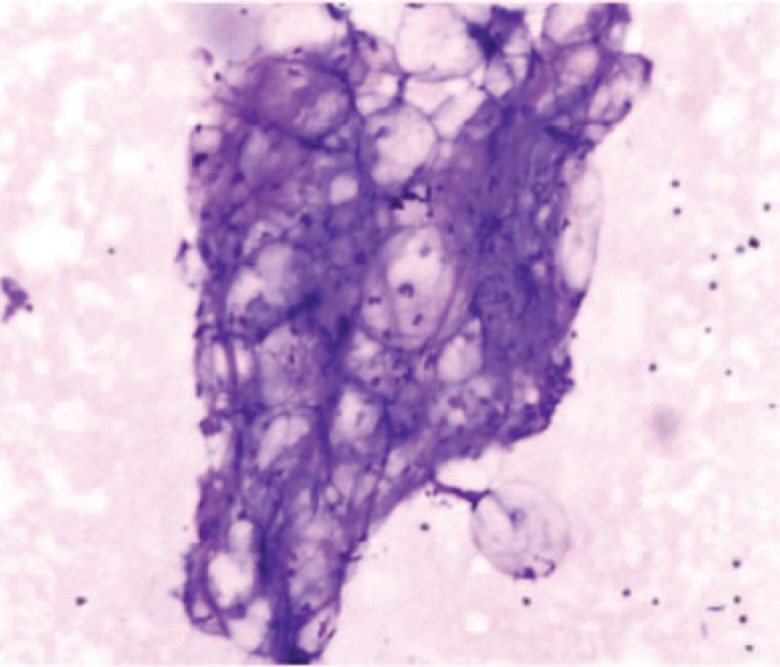
Cytosmear from FNA showing mature adipose tissue fragments (MGG 400x

All cytological smears yielded mature adipose tissue fragments. In view of ultra-sonographic finding and CT evaluation, a diagnosis of giant subcutaneous lipoma of neck was made (Figure 2). A complete removal of the mass was further advised. On surgery, a skin crease incision was made, and a large lobulated fatty mass was removed from the subcutaneous plane. The base was ligated and cut, and the mass was removed completely. A large flap of overlying skin was also removed. There was no localized lymphadenopathy or adherence to the surrounding tissues. 

**Fig. 3:**
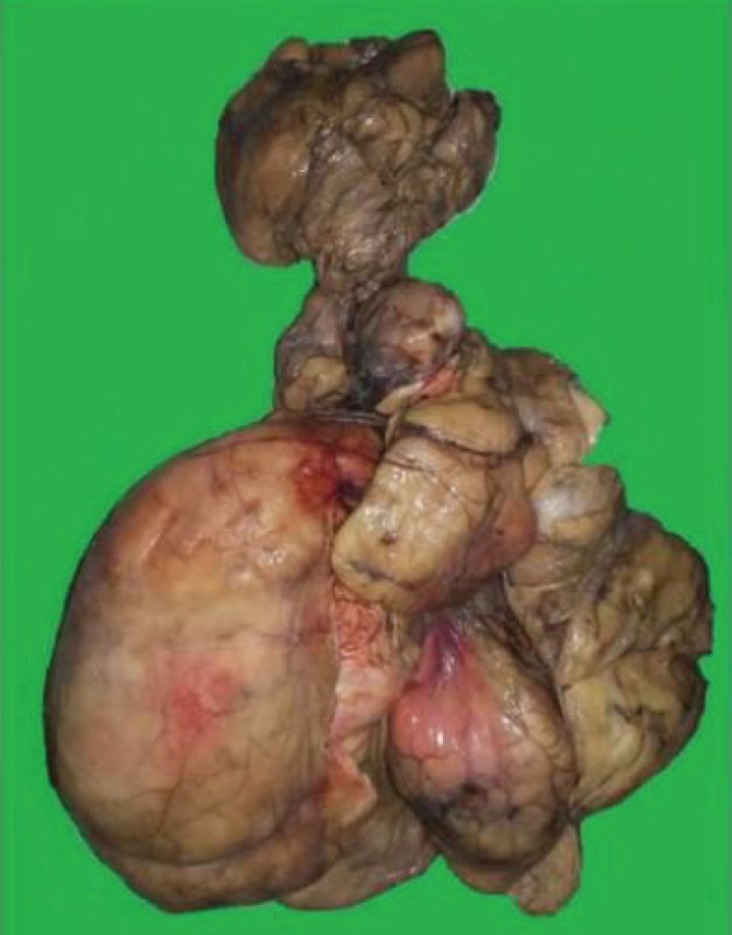
Gross photograph of the specimen measuring 32 cm in the longest diameter and weighing 2500 grams. Mass is yellow and lobulated in appearance

**Fig. 4:**
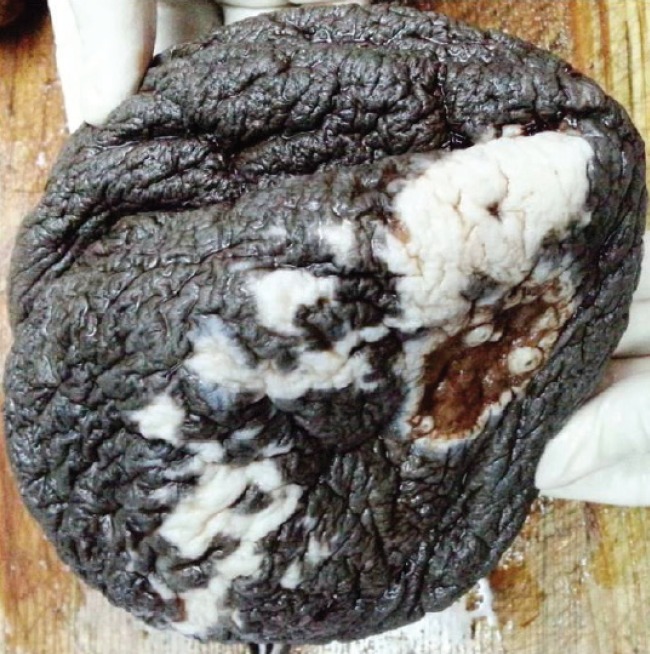
Gross photograph of the skin flap showing ulcerated surface

**Fig. 5:**
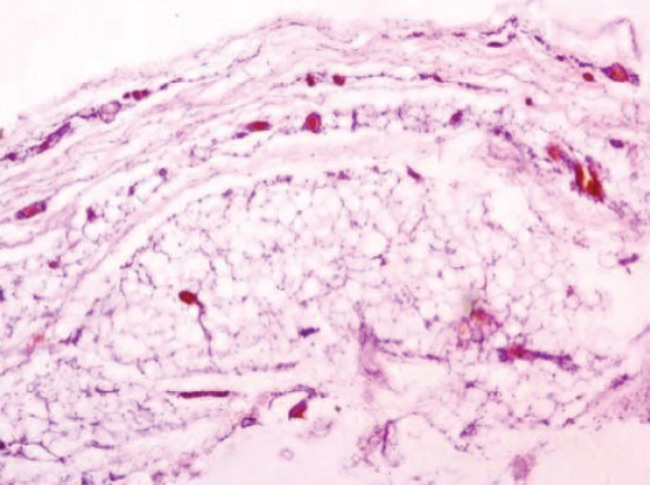
Microphotograph showing capsulated mature adipose tissue lesion (H&E 100x).

On gross examination, excised specimen was yellow in color and lobulated in appearance. It measured 32 cm in the longest diameter and weighed 2500 grams; parts of the thin capsule could be seen over the resected specimen (Figure 3). Overlying skin flap showed ulcerations (Figure 4). Exhaustive sampling of the specimen was done and on microscopy it showed capsulated mature adipose tissue with lobular appearance (Figure 5). There was no evidence of malignancy or heterologus element in multiple microsections studied. A final diagnosis of giant anterior neck lipoma with pressure ulcer was made.

## DISCUSSION

Lipoma is a benign tumour of mesenchymal origin that can occur anywhere in the body.^6^ Only 13% of all lipomas are located in the head and neck region of which posterior neck is the commonest site. Anterior neck lipomas are extremely rare.^7^^-^^9^ A few reported cases of anterior neck lipomas are all less than 16 cm in greatest dimension.^7^^-^^9^ Our patient had an exceptionally large lipoma (32 cms in greater dimension) in the anterior neck. Our patient was 70-year old male, though lipomas in this region commonly occur in women usually in the fourth and fifth decades.^10^

Owing to this location, they can be mistaken for thyroid masses and therefore, it was necessary to perform proper diagnostic tests to confirm the nature of the tumor and exclude possible communication with the thyroid. Comprehensive radiological evaluation performed in our case showed that the mass laid in front of the left thyroid lobe with no communication to the thyroid gland. Intra-operatively, lipomas may be seen as soft, yellow, shiny, smooth, mobile, encapsulated masses and occasionally may be lobulated. They are usually slow-growing nodules with the consistency of solid rubber. 

Microscopically, the lesions showed lobular growth of mature adipocytes and a fibrous capsule.^10^ Most lipomas pose no diagnostic problem. Rarely, lipomas can also become malignant. Hence, when presented with large or rapidly growing masses, especially of the head and neck region, one should rule out liposarcoma. De novo liposarcoma should also be kept in differential diagnosis.^11^^,^^12^ There is also an extremely rare^13^ possibility of having complications like bleeding pressure ulcer as was seen in our patient. However, we did not come across pressure ulcer in any of the anterior neck lipomas reported earlier. Also, improved diagnostic imaging techniques such as CT or magnetic resonance imaging (MRI) helped in the diagnosis of complex or unusual neck masses. 

Complete excision is the treatment of the choice and since they have a well-defined pseudo-capsule, dissection around these benign neoplasm is performed easily. Liposuction for such tumours has also been reported.^12^ To the best of our knowledge, we report the largest lipoma in the anterior neck region till date. Also, this is the first anterior neck lipoma with complications like bleeding pressure ulcer. Although the tumor is benign and surgery is completely curative, it is unfortunate to see patients presenting with such giant lipomas, carrying the huge brunt of psychological trauma for so long as a result of unawareness. 
